# The Effectiveness of Nanofat in the Management of Skin Scars: A Systematic Review

**DOI:** 10.1093/asjof/ojaf080

**Published:** 2025-07-02

**Authors:** Ghamdan Al-sabri, Sayed Alavi, Salem Alkaabi, Melvin Maningky, Tim Forouzanfar, Marco Helder

## Abstract

Nanofat is an adipose tissue product obtained through mechanical emulsification, preserving stromal vascular fraction and extracellular matrix. Nanofat has been primarily investigated in the contexts of skin scar repair. This systematic review assesses existing evidence on the effectiveness of nanofat for various skin scar types. In this systematic review, we aim to evaluate the effectiveness of nanofat injections in improving skin scars compared with controls, considering clinical, radiographical, histological, and histomorphometrical outcomes. This systematic review was conducted in accordance with Preferred Reporting Items for Systematic Reviews and Meta-Analyses guidelines. Databases including MEDLINE, Embase, and the Cochrane Library were searched from January 2013 through March 2025. Studies involving human patients receiving nanofat for scar treatment were included. Risk-of-bias assessments were performed by the Cochrane RoB 2 tool for randomized controlled trials (RCTs) and the NIH Quality Assessment Tool for Pre–Post Studies lacking control groups. Of 415 identified publications, 12 studies were included, comprising 5 RCTs and 7 pre–post intervention studies. Participant numbers ranged from 12 to 48. Most studies reported substantial short-term improvements in scar appearance, texture, and patient satisfaction. Histologically, findings indicated increased skin thickness, improved neovascularization, and collagen deposition. However, 2 high-quality RCTs showed no sustained significant benefits beyond 12 months. Few minor complications were documented; no serious adverse events occurred. Overall, nanofat injections appear to be an effective, minimally invasive treatment for skin scars, particularly in the short term. Nonetheless, the long-term benefits are less certain, underscoring the importance of conducting further research by standardized protocols and extended follow-up periods to confirm these findings.

**Level of Evidence**: 4 (Therapeutic): 
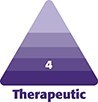

Harvested fat contains adipocytes, adipose stem cells (ASCs), extracellular matrix (ECM), endothelial cells, fibroblasts, and blood cells.^1^ The isolation of ASCs, or stromal vascular fraction (SVF), from harvested fat tissue can be achieved through chemical or mechanical methods. In the chemical approach, the fat is treated with a digesting enzyme to isolate the SVF-containing stem cells. However, this method has several disadvantages: it is time-consuming, requires specialized methodology and equipment, and must be performed by experienced laboratory technicians.^2^ Additionally, this process is generally considered costly owing to the requirement of clinical-grade products to prevent contamination.^3,4^ Furthermore, because chemical isolation of SVF significantly alters the original properties of fat tissue, many regulatory authorities consider it to be “more than minimal manipulation.”^5,6^ Because of these drawbacks, significant efforts have been made to develop enzyme-free techniques for isolating SVF. One of these new techniques is mechanical isolation, which involves mechanically breaking down the structural elements of adipose tissue.^7,8^ The outcome of this process is SVF with a preserved ECM structure.^9^ This matrix serves as physical support and scaffolding during grafting. The retained structural elements are thought to promote interactions that reduce cell death and increase graft retention.^10,11^

The end product of the mechanical technique contains cell debris, blood cells, and ECM, whereas the chemical technique results mainly in a pure cellular SVF.^12^ Alexander distinguished between cellular SVF (cSVF), which is the end product of the chemical method, and tissue SVF (tSVF), obtained from the mechanical method. Thus, the recommended term for the mechanically disrupted fat product is “tSVF,” and “cSVF” refers to the product obtained through chemical digestion of fat, in which the cellular components are isolated.^11^

The term “nanofat” was first introduced by Tonnard et al to describe an injectable product derived from a new mechanical technique to treat harvested lipoaspirate. Although this product is neither at the nanoscale nor does it contain viable fat cells, the term “nanofat” is still more commonly adopted than “tSVF.” The technique involves mechanical emulsification and filtration of lipoaspirate, resulting in a homogeneous and fine solution. Because of its smooth consistency, nanofat can be injected into patients through a small needle for rejuvenation purposes and scar treatment/prevention purposes.

The emulsification process involves squeezing the lipoaspirate 30 times between 2 syringes connected by a step-down diameter series of Luer-lock transfers (typically 2.4, 1.4, and 1.2 mm). After emulsification, the fat is filtered through a 400 to 600 μm filter to remove connective tissue fragments. The resulting injectable liquid is called nanofat. This technique is quick, easy, and inexpensive, making it an efficient way to convert a small amount of lipoaspirate into an injectable form that can be administered during the same surgical session.^9^

So far, nanofat has been applied in various clinical procedures, including skin rejuvenation, scar repair, management of vocal fold scar, and treatment of facial scleroderma.^9,13-26^

Nanofat has been primarily investigated in the contexts of skin scar repair. Therefore, in this study, we aim to review data from various studies that explored the application of nanofat in treating skin scars, with the goal of evaluating the effectiveness of these interventions.

## METHODS

This systematic review was conducted according to the guidelines of the Preferred Reporting Items for Systematic Reviews and Meta-Analyses (PRISMA).^27^

### Focus Question

The focus question for this review was formulated by the problem, intervention, comparison, and outcome (PICO) framework.

Problem (*P*): skin scar

Intervention (I): injecting skin scar with nanofat

Comparison (C): efficiency of nanofat injections vs control conditions (no injection or other fat grafting materials or treatment modalities)

Outcome (O): improvement in skin scars, as assessed with measurements of clinical, radiographical, or histological/histomorphometrical changes

Focus question: In human patients with skin scars, how effective is injecting nanofat compared with control conditions (no injection or other fat grafting materials) in improving skin scars as assessed by clinical, radiographical, or histological/histomorphometrical changes?

### Eligibility Criteria

Before proceeding with the search, the inclusion and exclusion criteria were established. Publications had to meet the following criteria to be considered for inclusion in this systematic review: investigation of nanofat in the treatment of skin scars, involvement of human participants, and publication in English between January 1, 2013, and March 12, 2025. Publications were excluded if they did not meet these requirements. Additionally, publications were excluded if nanofat was not the main focus of the research, if there was inadequate data, or if the preparation protocol of nanofat was not adequately described.

### Search Strategy

Our systematic review was conducted according to the guidelines of the PRISMA in MEDLINE, Embase, and Cochrane library on March 12, 2025. To identify additional studies, we conducted backward-reference list checks as part of the identification process. The search adhered to the PICO protocol, with search terms encompassing: “nanofat,” “nano-fat,” “lipoconcentrate,” “Stromal vascular fraction,” “t-VSF,” and “skin scar” (Supplemental Table 1). Two reviewers (Ghamdan Al-sabri (G.A.) and Sayed Alavi (S.A.)) conducted the search by the aforementioned keywords. At first, titles and abstracts were screened, followed by a thorough examination of the full texts of potentially eligible studies based on the predefined eligibility criteria. Both reviewers independently validated the extracted data from the included studies. In case of any disagreement, a resolution was sought by consulting a third reviewer (M.H.).

### Data-Charting Process

An Excel data sheet (Microsoft Corporation, Redmond, WA) was created to identify the data variables to extract. The extracted information included authors and publication year, sample size, study design, nanofat preparation protocol, type of scar studied, fat harvesting method, outcome evaluation method, complications, follow-up period, and findings.

The reviewers independently documented the extracted data from the included studies and recorded it in the Excel datasheet. Any disagreements were resolved through discussions between the 2 reviewers.

### Risk of Bias Assessment

In this review, we assessed the risk of bias (RoB) in the included studies by 2 different tools. Five randomized clinical trials (RCTs) were evaluated by the Cochrane Risk-of-Bias Tool for Randomized Trials (RoB 2), while the remaining 7 studies, which were pre–post studies without a control group, were assessed by the National Institutes of Health (NIH) Quality Assessment Tool for Before–After (Pre–Post) Studies With No Control Group, given the nature of these studies. The risk-of-bias assessments for each study were conducted independently by 2 researchers. Any disagreements were resolved through discussion.

### Cochrane Risk-of-Bias Tool for Randomized Trials (RoB 2)

In this systematic review, we assessed the RoB in the included RCTs by the RoB 2 tool: A Revised Tool for Assessing Risk of Bias in Randomized Trials. This tool was developed by the Cochrane Collaboration to provide a rigorous and comprehensive framework for evaluating bias in randomized trials. The revised version of the original RoB tool introduces a structured, domain-specific approach designed to enhance transparency and consistency in bias assessments across different studies.

RoB 2 is divided into 5 key domains essential for evaluating the internal validity of randomized trials: (1) bias arising from the randomization process, (2) bias owing to deviations from intended interventions, (3) bias owing to missing outcome data, (4) bias in the measurement of outcomes, and (5) bias in the selection of the reported results. Each domain is rated as “low risk,” “some concerns,” or “high risk,” based on the study design, conduct, and reporting.^28^

After evaluating all the domains, we generated an overall RoB judgment for each study, reflecting its overall level of bias.

### NIH Quality Assessment Tool for Pre–Post Studies Without a Control Group

For the assessment of RoB in the pre–post studies without a control group included in this review, we employed the NIH Quality Assessment Tool for Before–After (Pre–Post) Studies With No Control Group. This tool is specifically designed for studies lacking a control group and focuses on evaluating outcomes before and after an intervention. It provides a structured framework for assessing the quality of such studies by considering critical elements such as study design, sample size, data collection methods, and outcome measurement.

The tool composes 12 questions that guide the assessment of the study's internal validity. These questions evaluate various factors, including the clarity of the study objectives, the selection of participants, the accuracy and reliability of outcome measures, and the handling of potential confounding factors (Supplemental Table 2). Each question is rated as “Yes,” “No,” “Not Applicable,” or “Cannot Determine,” based on the information provided in the study.^29^

The overall RoB for each study was determined based on a structured judgment of the responses to individual criteria. Studies were rated as having a low, moderate, or high RoB. A study was considered to have a low RoB if most criteria were answered with “Yes,” particularly those related to critical aspects such as clear research questions, defined eligibility criteria, valid and reliable outcome measures, appropriate statistical analyses, and sufficient follow-up. A moderate RoB was assigned when some important criteria were marked “No” or “Can't Determine,” indicating the presence of methodological concerns that may affect the results but are unlikely to invalidate them. Studies with a high RoB had several important criteria marked “No,” indicating major methodological flaws, which may seriously compromise the validity of the findings.

## RESULTS

### Search Results

**Figure 1.**
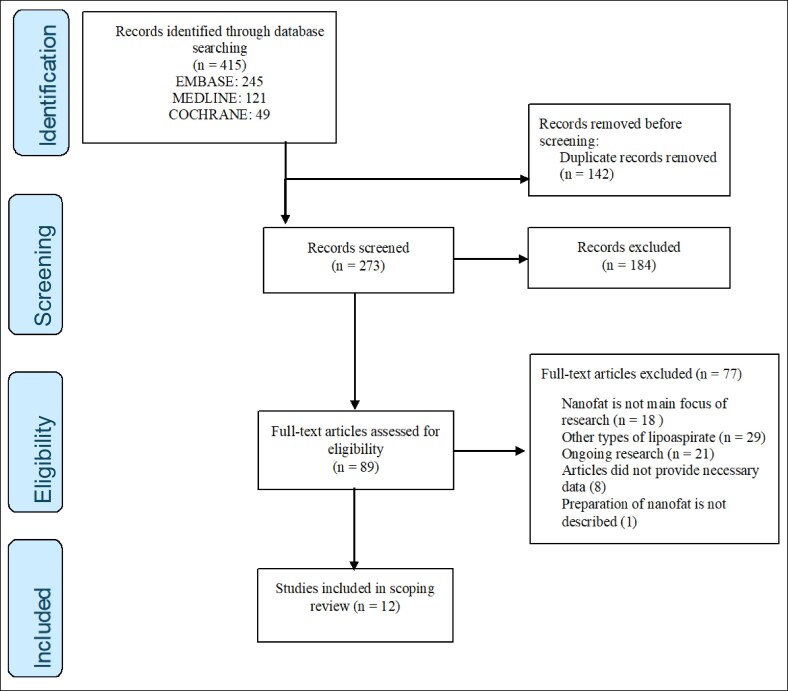
Preferred Reporting Items for Systematic Reviews and Meta-Analyses flow diagram article selection. Flow diagram of search and study selection on the basis of the Preferred Reporting Items for Systematic Reviews and Meta-Analyses (PRIMSA) guidelines.

The primary online database search yielded a total of 415 publications, distributed as follows: 245 in Embase, 121 in MEDLINE, and 49 in Cochrane Central (Figure 1). Before further screening, 142 publications were excluded owing to duplication, leaving 273 publications for further assessment. After reviewing titles and abstracts, 184 articles were excluded. Subsequent screening of the full texts resulted in the exclusion of 77 publications based on predetermined inclusion and exclusion criteria. Based on these criteria, a total of 12 publications were included in our review. A summary of the included studies is presented in Table 1, and a more detailed overview is presented in Supplemental Table 3.

**Table 1.** Summary Characteristics of Included Studies on Nanofat Treatment for Skin Scars

Study Author (Year)	Study Design	Sample Size	Scar Type	Nanofat Preparation	Follow-up period
Tenna et al (2017)^20^	Prospective comparative	30	Acne scars	Centrifugation, emulsification, + PRP	6 months
Gentile et al (2017)^15^	Prospective comparative	43	Burn/posttraumatic scars	Mechanical emulsification; multiple variants	12 months
Uyulmaz et al (2018)^14^	Retrospective	40	Mixed	Emulsification, filtration	3 months
Gu et al (2018)^16^	Prospective comparative	20 (25 sites)	Atrophic scars	Centrifugation, emulsification, second centrifugation	Not specified
Jan et al (2019)^17^	Prospective comparative	48	Postburn scars	Emulsification only	6 months
Bhooshan et al (2018)^13^	Prospective comparative	34	Mixed (79.4% post traumatic)	Emulsification, gauze filtration	3 months
Huang et al (2021)^24^	Retrospective	44	Depressed facial scars	Centrifugation, emulsification, filtration	12 months
Rageh et al (2021)^19^	Prospective comparative	30	Mixed (63.3% post traumatic)	Centrifugation, emulsification, filtration	6 months
Kemaloğlu et al (2021)^18^	Prospective comparative	45	Breast reduction scars	Centrifugation, emulsification, filtration	6 months
van Dongen et al (2022)^21^	Randomized controlled trial (RCT)	34	Breast reduction scars	Centrifugation, emulsification, second centrifugation	12 months
Ramaut et al (2024)^22^	Split-scar RCT	12	Post surgical (abdominoplasty)	Centrifugation, emulsification, filtration	12 months
Rageh et al (2025)^23^	Prospective comparative	30	Postburn scars	Centrifugation, emulsification, filtration	4 months

Ten of these studies were prospective, and only 2 studies were conducted retrospectively, with sample sizes ranging from 12 to 48 samples (average: 34 samples).^13-24^

### Nanofat Preparation

The techniques employed for nanofat preparation varied significantly across the included studies. Three studies followed the Tonnard method, which involves direct emulsification of lipoaspirate followed by filtration.^9,13-15^ In 1 study, nanofat was prepared solely by emulsifying lipoaspirate without subsequent filtration.^17^ In 5 studies, the lipoaspirate was first centrifuged at a specific speed and duration before undergoing emulsification and filtration.^18,19,22-24^ Tenna et al also centrifuged the lipoaspirate before emulsification but omitted the filtration step; instead, they mixed the resulting nanofat with platelet-rich plasma (PRP) prior to application.^20^ In 2 other studies, a first centrifugation was followed by emulsification, and a second centrifugation replaced the filtration process.^16,21^ It is worth noting that the centrifugation force varied among the studies.

In addition to the classic nanofat prepared as described by Tonnard, Gentile et al prepared 3 other nanofat modifications: supercharge-modified nanofat, evo-modified nanofat, and centrifuge-modified nanofat. Supercharge-modified nanofat was prepared by mixing emulsified fat (30 passes) with centrifuged and filtered lipoaspirate. Evo-modified nanofat was produced by slow centrifugation of lipoaspirate (80 rpm per minute) followed by emulsification (30 passes). Centrifuge-modified nanofat was obtained by directly centrifuging lipoaspirate without an initial filtration and then emulsifying it (30 passes).^15^

### Type of Skin Scar

The effect of nanofat was evaluated on different types of scars across the included studies. Two studies focused solely on postburn scars involving a total of 78 patients, and 2 studies investigated its impact on at total of 64 patients with atrophic scars.^16,17,23,24^ Three studies examined its application in postsurgical scars covering 91 patients, and only 1 study assessed its effect on acne scars (30 patients).^18,20-22^ Additionally, 4 studies explored nanofat treatment across various types of scars.^13-15,19^

### Evaluation Methods

The evaluation methods adopted across the studies varied considerably. Objective assessments included measurements such as skin thickness by ultrasound, as reported by Tenna et al, and histological analysis, which was performed in 5 studies.^15,16,19-22^ In 1 study, Rageh et al utilized the Antera 3-dimensional (3D) (Miravex Limited, Dublin, Ireland) skin analysis camera to evaluate changes in skin texture and pigmentation.

Subjective assessments also differed among the studies. A total of 4 studies relied on physician evaluations, 2 studies employed patient satisfaction tools such as the FACE-Q questionnaire, and 1 study applied simple verbal rating scales.^14,15,20,21,24^ The Patient and Observer Scar Assessment Scale (POSAS) was employed in 5 studies, while the Vancouver Scar Scale (VSS) was applied in 3 studies.^13,16-19,21-23^ Additionally, the Visual Analog Scale (VAS) was employed to capture patient-reported outcomes in 1 study.^18^

In summary, the studies employed a combination of quantitative and qualitative evaluation methods, emphasizing the value of both objective measurements and patient-reported experiences in assessing the effectiveness of nanofat treatment for scars.

### Complications

In 5 studies, no complications were reported.^14,15,18,21,23^ Another 5 studies documented only minor complications. Rageh et al reported bruising in 13.3% of patients, edema in 16.7%, erythema in 10%, and hyperpigmentation in 3.3%. Huang et al reported temporary erythema in 93% of patients, pigmentation in 2.3%, and blistering in 4.5%. Bhooshan et al noted 2 cases (5.9%) of itching, redness, and excoriation. Jan et al documented mild edema in 62.5% of patients, without bruising, cysts, or granulomas. In 2 studies, complications were not mentioned.^16,20^ Notably, no major complications were reported in any of the included studies.

### Effect of Nanofat

Tenna et al conducted a study to assess the effectiveness of nanofat mixed with PRP with or without CO_2_ laser resurfacing for treating atrophic facial scars. Thirty patients underwent treatment involving nanofat and PRP infiltration, with fractional CO_2_ laser resurfacing immediately performed in 15 of these patients. Comparing skin thickness, as measured by ultrasound scan, between the 2 groups revealed no significant difference. Patient satisfaction assessments did not indicate any advantages in skin resurfacing with CO_2_ laser following treatment with nanofat and PRP. However, evaluating the change in skin thickness before and after nanofat and PRP infiltration demonstrated a significant increase in skin thickness. Furthermore, the study demonstrated a high level of patient satisfaction after the application of nanofat and PRP.^20^

Gentile et al investigated the effect of 3 different modified nanofat products—supercharge-modified nanofat, evo-modified nanofat, and centrifuge-modified nanofat—on postburn and posttraumatic scars and compared them with classic nanofat prepared as described by Tonnard. To assess skin scar improvement, they applied a patient self-evaluation questionnaire, an operator evaluation questionnaire, and preoperative and postoperative biopsies for histological examination. The results from patient self-evaluation and operator evaluation indicated that supercharge-modified nanofat offered the best scar improvement, followed by evo- and centrifuge-modified nanofat, and finally classic nanofat. Histopathological examination of preoperative and postoperative biopsies from all groups demonstrated a shift toward more skin regeneration with normal epithelium and deposition of collagen fibers and vessels. Similarly, total epidermal and dermal thickness postoperatively showed a significant increase in skin thickness compared with preoperative levels in all groups. However, the difference in skin thickness and regeneration among the different nanofat subgroups was not significant.^15^

In a retrospective study, Uyulmaz et al investigated the effect of nanofat on skin scars in a group of 40 patients. Three independent physicians evaluated preoperative and postoperative photographs and found a significant improvement in the appearance of scars: 74% were deemed good, 18% were considered satisfactory, and only 8% exhibited no change. Moreover, 92% of patients showed a high level of satisfaction with their outcomes.^14^

Gu et al investigated the effect of nanofat in 25 atrophic facial scars (20 patients) of diverse etiology. They compared the POSAS preoperatively and 6 months postoperatively to assess clinical outcomes. All variables of the POSAS, as measured by both patients and physicians, demonstrated statistically significant improvement, except for pain, itching, and vascularization. Histological examination revealed an increase in melanin average optical density, with no observed changes in elastic fibers. Immunostaining conducted 6 months after intervention showed the presence of sebaceous and sweat glands, which were undetectable preoperatively. Overall, they found nanofat to significantly improve the aesthetics of atrophic facial scars.^16^

Jan et al investigated the effect of unfiltered nanofat on postburn facial scars. Forty-eight patients received unfiltered nanofat in which preoperative and postoperative scar POSAS scores were compared. Nanofat was prepared following the Tonnard preparation technique but without the final step, filtration. After treatment with nanofat, the observer section of POSAS showed that there was a significant improvement in scar quality, especially in pigmentation and pliability parameters. However, scar thickness and relief showed insignificant improvement. On the contrary, the patient section of the POSAS showed a significant improvement in all parameters. Overall, they found that unfiltered nanofat appears to have a promising therapeutic effect in postburn facial scars.^17^

In a prospective observational study, Bhooshan et al investigated the effect of nanofat on 34 patients with facial scars of various etiologies; 27 had posttraumatic scars, 5 had postburn scars, and 2 had postinflammatory scars. They treated these patients with nanofat produced according to the Tonnard protocol. Pre- and 3-month postoperative POSAS scores were compared to evaluate the effectiveness of nanofat. They found that all patient and observer parameters of POSAS showed significant improvement. They concluded that nanofat improves the symptoms as well as the texture of facial scars and provides a promising modality in the treatment of extensive facial burn scarring when other modalities are not applicable.^13^

In a retrospective study, Huang et al treated 44 patients who suffered from depressed facial scars with nanofat and applied the FACE-Q scale as a tool to evaluate the effectiveness of this treatment. Three plastic surgeons evaluated the outcomes by comparing preoperative and postoperative photographs. The harvested fat was first centrifuged at 2000 rpm for 3 min before being emulsified and filtered according to the Tonnard preparation technique. They compared patients who had completed the treatment more than a year ago with those who had done so within a year. Those who had completed the treatment more than a year ago demonstrated significantly higher satisfaction with their decision to undergo this treatment compared with those who had the intervention within a year. Conversely, patients who completed the therapy within a year demonstrated significantly higher satisfaction with social function compared with those who finished the treatment more than a year ago. However, satisfaction with the appearance aspect of the FACE-Q scale did not show any significant difference between the 2 subgroups. Preoperative and postoperative photographs evaluated by surgeons showed a significant improvement in scarring (30% of cases showed complete healing, 41% obvious improvement, and 20% effective treatment. Only 9% of cases showed no change after therapy). The authors concluded that nanofat can provide a promising modality in the management of depressed facial scars.^24^

In their prospective study, Rageh et al investigated the effectiveness of nanofat injections on facial scars. They treated 30 patients with facial scars by nanofat. Harvested adipose tissue was first centrifuged at 1006 *g* for 3 min before being emulsified and filtered. The outcomes after 6 months were evaluated by means of the VSS and a histological comparison between preoperative and postoperative biopsies. The height and pliability aspects of the VSS showed a significant improvement after nanofat injections. However, vascularity and pigmentation aspects failed to show a statistically significant difference. The histological assessment showed a significant improvement in epidermal thickness 6 months after nanofat treatment. Neovascularization, collagen, and elastic fiber thickness were also significantly improved. In contrast, melanocyte staining showed no statistically significant difference. Overall, nanofat was shown to be an effective modality for managing scars of different etiologies. They concluded that nanofat improves the symptoms as well as the texture of facial scars and provides a promising modality in the treatment of extensive facial burns scarring when other modalities are not applicable.^19^

In a prospective study, Kemaloğlu et al investigated the effect of nanofat in treating postsurgical scars after breast reduction. They divided 45 patients into 3 groups: a control group that did not receive any tissue grafting with breast reduction surgery, a second group that received fat injections, and a third group that received a combination of fat and nanofat grafting. The nanofat was prepared by centrifuging lipoaspirate at 3000 rpm (1200 *g*) for 3 min before emulsification (30 passes) and filtration over a sterile nylon cloth. To evaluate the outcomes after 6 months, they applied the VSS, completed by independent blinded reviewers, and a VAS, completed by the patients themselves. They found that, apart from scar height, all VSS scores in both the fat and fat–nanofat groups exhibited significant reductions compared with the control group. In the comparison between the fat and fat–nanofat groups, pigmentation scores were significantly lower in the fat–nanofat group. There was no significant difference in vascularization, pliability, and height scores between the fat and fat–nanofat groups. Regarding the VAS, the fat and fat–nanofat groups scored lower than the control group, but there was no statistical difference between the fat and fat–nanofat groups. They concluded that simultaneous fat and nanofat grafting has beneficial effects on scars in breast reduction patients and should be considered a safe alternative to other wound intervention therapies.^18^

Van Dongen et al investigated the effect of nanofat on postsurgical scar formation. In their trial, 34 patients who underwent mammaplasty received nanofat graft simultaneously in 1 breast, whereas the contralateral breast received a placebo injection to serve as a control. They employed the POSAS, biopsies, and photographs to evaluate the effect of nanofat at 6 and 12 months postoperatively. According to POSAS, there was a significant improvement in scar appearance on the nanofat side compared with the placebo side at 6 months postoperatively. However, the difference was not significant after 12 months. Furthermore, histological and photographic evaluations did not reveal a significant difference between the nanofat and placebo sides at any of the time points studied. In summary, nanofat injection improved wound healing and minimized scar formation at 6 months postoperatively, without any significant advantage noticed at 12 months. Their results indicated that nanofat may only work as an accelerator of wound healing and scar formation.^21^

In their randomized, double-blind, split-scar controlled clinical trial, Ramaut et al evaluated the effect of nanofat infiltration on early scar maturation. Twelve patients undergoing abdominoplasty had nanofat injected into 1 side of the surgical scar, and the other side served as a control. Scar assessment was performed at 3, 6, and 12 months by the POSAS, spectrophotometry (erythema and pigmentation indices), and histological analysis. Nanofat infiltration was found to promote early clinical scar maturation, as reported by both patients and observers, but it did not lead to a significant improvement in the final scar appearance. Spectrophotometric analysis showed a reduction in erythema, indicating enhanced early scar maturation; however, this difference was no longer evident after 1 year. Furthermore, histological examination of tissue biopsies at the 8-month follow-up found no significant differences in epidermal or dermal thickness, elastic fiber content, or collagen structure between treated and control scars, suggesting that nanofat's effects may be limited to the early stages of scar healing.^22^

In their prospective study, Rageh et al evaluated the efficacy and safety of nanofat injections for the management of postburn scars by both subjective and objective assessment methods. Thirty patients underwent a single session of liposuction, followed by nanofat preparation and injection into their scar tissue. The treatment outcomes were assessed after 4 months by the VSS and Antera 3D skin imaging. The results showed statistically significant improvements in scar height, color, vascularity, and pliability, with the total VSS scores decreasing significantly post treatment. The Antera 3D analysis revealed significant reductions in scar indentations, erythema, and pigmentation scores. No side effects other than mild pain at the injection site were reported. The authors concluded that nanofat injections are a safe and effective treatment for postburn scars, improving both the aesthetic and functional aspects of scarring with minimal complications.^23^

### Risk of Bias

The risk of bias for the included studies was assessed by 2 tools: the Cochrane Risk of Bias Tool (RoB 2) for RCTs and the NIH Quality Assessment Tool for Pre–Post Studies Without a Control Group.

### Cochrane Risk of Bias Tool (RoB 2)

The RoB for the 5 studies included in the analysis was assessed by the Cochrane Risk of Bias 2 (RoB 2) tool.

Two studies (van Dongen et al and Ramaut et al) were assessed as having a low RoB across all domains.^21,22^ Three studies (Kemaloğlu et al, Gentile et al, and Tenna et al) were judged to have a high RoB overall.^15,18,20^ These studies showed concerns primarily in the bias owing to deviations from intended interventions domain, rated as high in all 3 studies. Additionally, Kemaloğlu et al had a moderate risk in the randomization process, whereas Gentile et al and Tenna et al had a high RoB in outcome measurement.

**Figure 2.**
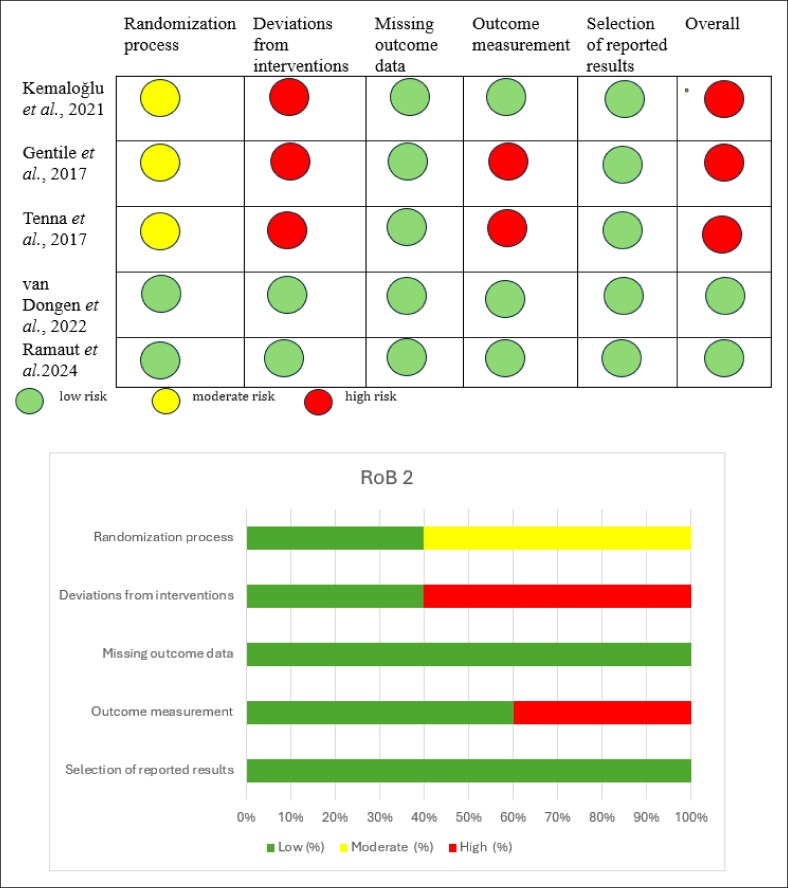
Risk-of-bias assessment of randomized controlled trials by the Cochrane RoB 2 Tool. Risk-of-bias assessment of the included studies by the Cochrane Risk of Bias 2 (RoB 2) tool. Two studies showed a low risk of bias across all domains. In contrast, 3 studies were rated as a high risk of bias, mainly owing to deviations from intended interventions and concerns in outcome measurement or randomization.

In contrast, van Dongen et al and Ramaut et al demonstrated a low RoB across all assessed domains, indicating higher methodological rigor compared with the other studies (Figure 2).

### NIH Quality Assessment Tool

**Figure 3.**
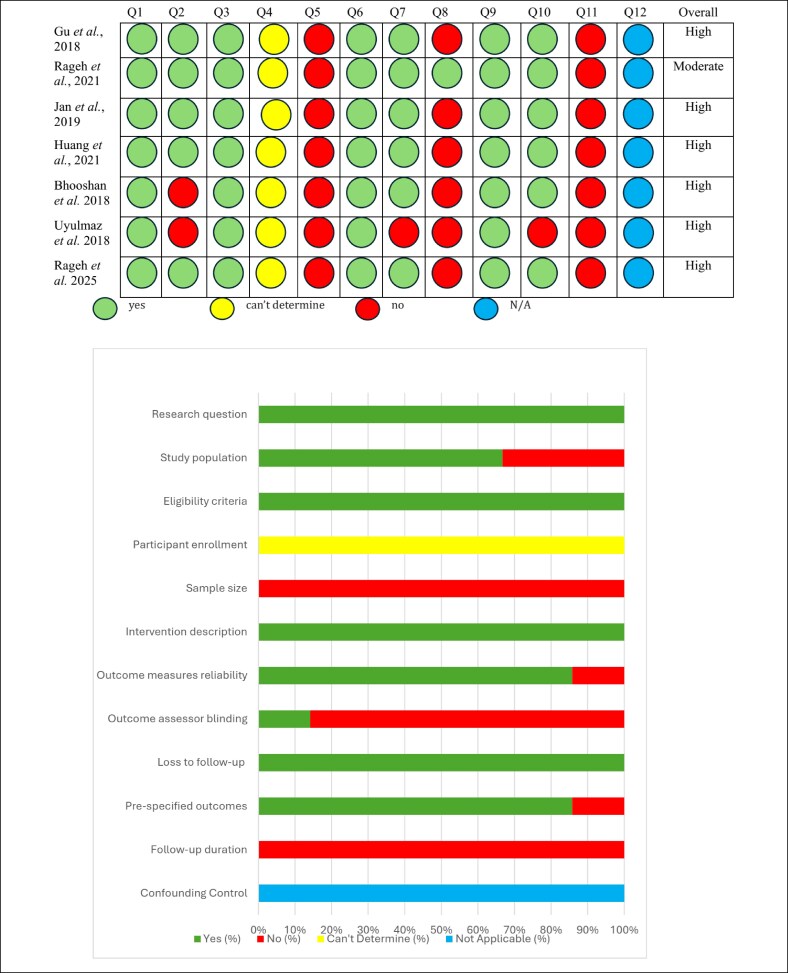
Risk-of-bias assessment using NIH Quality Assessment Tool. Quality assessment of the 7 pre–post studies by the NIH Quality Assessment Tool for Before–After Studies. One study was rated as a moderate risk of bias, while the remaining 6 were classified as a high risk of bias. Common limitations included inadequate sample size, lack of blinding, and absence of long-term outcome measurement. Most studies clearly described their interventions and outcomes and applied appropriate statistical analyses.

The quality of the 7 pre–post studies included in this review was assessed by the NIH Quality Assessment Tool for Before–After (Pre–Post) Studies. Six studies were classified as having a high RoB, while 1 study was rated as a moderate RoB.^13,14,16,17,19,23,24^ Across all studies, the study question and eligibility criteria were clearly stated (Q1, Q2). However, sample size sufficiency (Q5) was consistently rated “no” across all studies. Blinding of outcome assessors (Q8) was also not implemented in most studies, increasing the potential for measurement bias. Furthermore, long-term outcome measurements (Q11) were generally not taken. Despite these limitations, most studies clearly described their intervention (Q6) and outcome measures (Q7), except for Uyulmaz et al, which was marked as “no” for Q7.^14^ All studies appropriately applied statistical methods to examine changes in outcome measures from before to after the intervention, and all except Uyulmaz et al reported *P*-values for pre-to-post changes (Q10).^14^ Loss to follow-up (Q9) was generally low across the studies (Figure 3).

Overall, 1 study was rated as a moderate RoB, while the remaining 6 were classified as a high RoB.

## DISCUSSION

The primary objective of this systematic review was to evaluate the efficacy of nanofat treatment in the management of various skin scars. The results from the 12 included studies offer comprehensive insights into the therapeutic potential and limitations of nanofat, while also highlighting critical methodological aspects influencing outcome reliability.

Across all studies, nanofat therapy consistently showed beneficial effects on scar quality, especially during early postoperative periods. However, critical nuances emerged from high-quality RCTs. Specifically, van Dongen et al and Ramaut et al, which were the only studies identified as having a low RoB and robust methodological designs, demonstrated significant improvements in scar appearance at 6 months posttreatment according to the POSAS. Importantly, both studies showed that these positive effects diminished by 12 months, suggesting that nanofat primarily accelerates early scar maturation without significantly improving the final scar appearance. These findings underscore the necessity of longer-term evaluations, as short-term assessments might overestimate the sustained clinical efficacy of nanofat. However, it should be taken into account that both studies applied nanofat in a postsurgical (preventive) setting, in contrast to the other studies included in this review. The only other study applying the nanofat in this manner was the study of Kemaloğlu et al, but their evaluation was only performed at a follow-up time of 6 months.^18^

Preparation methods of nanofat varied notably among studies, influencing both biological characteristics and clinical outcomes. Most studies utilized variations of the emulsification and filtration protocol initially described by Tonnard et al, with many incorporating additional centrifugation steps prior to emulsification.^9^ Tenna et al uniquely implemented an adjunctive step involving PRP.^20^ Gentile et al experimented with multiple centrifugation-based nanofat variants and reported enhanced scar improvement with supercharge-modified nanofat compared with other variations. However, these modifications did not yield statistically significant differences in skin thickness or cellular regeneration, suggesting that the primary mechanism of action remains consistent across these approaches.^15^

Regarding scar types, nanofat appeared effective across a spectrum, including atrophic, post burn, acne, and postsurgical scars. Improvements were observed regardless of etiology, supporting nanofat's broad applicability. For example, atrophic facial scars exhibited marked aesthetic and histological improvements, and postburn scars significantly benefited from improved pliability and pigmentation.^16,17,23^ This suggests a broad spectrum of applicability; however, standardized treatment protocols per scar type are necessary to enhance reproducibility.

Evaluation methods varied considerably, combining objective measures (ultrasound skin thickness, histological analyses, spectrophotometry, and 3D skin analysis camera) and subjective assessments (patient satisfaction, POSAS, and VSS). Although variability complicates direct comparisons, the frequent application of validated scales such as POSAS and VSS offers reliability in demonstrating meaningful improvements postnanofat treatment. Objective measurements, however, sometimes contradicted subjective findings, as demonstrated by van Dongen et al, highlighting the complexity of scar assessment and the need for multidimensional evaluation approaches.^21^

Regarding safety, nanofat application consistently demonstrated an excellent safety profile, with only minor complications reported, including erythema, edema, bruising, and mild hyperpigmentation. Importantly, no severe or long-lasting adverse effects were observed, supporting nanofat as a safe therapeutic option. Nonetheless, systematic documentation of long-term complications remains limited and warrants further investigation.

The risk-of-bias assessment revealed substantial methodological limitations in many studies. The highest methodological rigor was evident in 2 RCTs; both have a low RoB.^21,22^ Conversely, other RCTs showed a high RoB primarily owing to deviations from intended interventions and insufficient blinding of outcomes.^15,18,20^ Before–after studies also exhibited high risk owing to small sample sizes and inadequate blinding. These limitations indicate that future research should prioritize methodological rigor, adequate sample sizes, and robust blinding procedures to strengthen evidence reliability.

This review has several limitations primarily owing to significant heterogeneity among included studies in terms of design, sample sizes, nanofat preparation methods, scar types, and evaluation tools. The variability complicates drawing definitive conclusions. Thus, future research must emphasize standardizing nanofat preparation, adopting uniform and comprehensive scar evaluation methods, and conducting longer-term follow-up studies to assess durability and long-term safety. Furthermore, future studies should replicate the robust methodological approaches exemplified by van Dongen et al and Ramaut et al to minimize bias and enhance the reliability of clinical outcomes.

## CONCLUSIONS

Nanofat demonstrates promising potential as a safe and minimally invasive treatment for improving the appearance and texture of various skin scars. Although short-term benefits are consistently reported across studies, the current evidence, particularly from high-quality trials, suggests that these effects may not persist long term. Standardized protocols, longer follow-up, and rigorous study designs are essential to fully establish nanofat's role in scar management and to optimize its therapeutic outcomes.

## Supplementary Material

ojaf080_Supplementary_Data
