# Supplementary material for: The Effectiveness of Nanofat in the Management of Skin Scars: A Systematic Review
Source: Aesthet Surg J Open Forum. 2025 Jul 2;7:ojaf080. doi: 10.1093/asjof/ojaf080 (PMC12343075; doi:10.1093/asjof/ojaf080)
Supplement: ojaf080_Supplementary_Data [file ojaf080_supplementary_data.zip › Table_S1.docx]

**Supplementary Table S1.**The search strategies for PubMed and EMBASE

| **PubMed** | ((Nanofat [tw] OR Microfat [tw] Nano-fat [tw] OR Micro-fat [tw] Lipoconcentrate [tw] OR "Stromal vascular fraction" [tw] OR SVF [tw] OR "Tissue- stromal vascular fraction" [tw] OR t-SVF [tw]) OR (Nanofat [mh])) AND (("Skin scar" [tw] OR Scar* [tw]) OR (Scar [mh])) |
| --- | --- |
| **Embase** | ('nanofat'/exp OR nanofat OR 'nano-fat' OR 'micro fat' OR lipoconcentrate OR 'stromal vascular fraction'/exp OR 'stromal vascular fraction' OR svf OR 'tissue-stromal vascular fraction' OR 't-svf') AND ('skin scar'/exp OR 'skin scar' OR scar* OR 'scar'/exp OR scar) |
| **Cochrane Library** | ((scar OR scarring OR skin scar) in All Text AND (nanofat OR Microfat OR nano-fat OR micro-fat OR Lipoconcentrate OR "Stromal vascular fraction" OR "SVF" OR "Tissue- stromal vascular fraction" OR "t-SVF") in All Text)) |
