# Supplementary material for: The Effectiveness of Nanofat in the Management of Skin Scars: A Systematic Review
Source: Aesthet Surg J Open Forum. 2025 Jul 2;7:ojaf080. doi: 10.1093/asjof/ojaf080 (PMC12343075; doi:10.1093/asjof/ojaf080)
Supplement: ojaf080_Supplementary_Data [file ojaf080_supplementary_data.zip › Table_S2.docx]

**Supplementary Table S2.** NIH Quality Assessment Tool for Pre-Post Studies Without a Control Group

| **#** | **Question** | **Yes** | **Not determined** | **No** | **N/A** |
| --- | --- | --- | --- | --- | --- |
|  | Was the study question or objective clearly stated? |  |  |  |  |
|  | Were eligibility/selection criteria for the study population clearly described? |  |  |  |  |
|  | Were the participants representative of those who would be eligible for the intervention in the general or clinical population? |  |  |  |  |
|  | Were all eligible participants that met the study entry criteria enrolled? |  |  |  |  |
|  | Was the sample size sufficiently large to provide confidence in the findings? |  |  |  |  |
|  | Were the intervention(s) clearly described? |  |  |  |  |
|  | Were the outcome measures clearly defined, valid, reliable, and implemented consistently across all study participants? |  |  |  |  |
|  | Were the people assessing the outcomes blinded to the participants' exposures/interventions? |  |  |  |  |
|  | Was the loss to follow-up after baseline 20% or less? |  |  |  |  |
|  | Did the statistical methods examine changes in outcome measures from before to after the intervention? Were statistical tests used that provided p-values for the pre-to-post changes? |  |  |  |  |
|  | Were outcome measures taken multiple times before and after the intervention (i.e., was an intervention period long enough to see an effect)? |  |  |  |  |
|  | If the intervention was conducted at a group level, did the statistical analysis take into account individual-level data to avoid confounding? |  |  |  |  |
