# Supplementary material for: The Effectiveness of Nanofat in the Management of Skin Scars: A Systematic Review
Source: Aesthet Surg J Open Forum. 2025 Jul 2;7:ojaf080. doi: 10.1093/asjof/ojaf080 (PMC12343075; doi:10.1093/asjof/ojaf080)
Supplement: ojaf080_Supplementary_Data [file ojaf080_supplementary_data.zip › Table_S3.docx]

**Supplementary Table S3.**  The charted data for each individual source of evidence

| study | Sample size | Type of study & design | Nanofat preparation | Type of scar | Harvesting the fat | Evaluation method | complications | Follow up period | findings |
| --- | --- | --- | --- | --- | --- | --- | --- | --- | --- |
| 1) Tenna *et al.* 2017 | 30 | - Group 1 underwent a fractional CO2 laser resurfacing after nanofat & PRP infiltration - Group 2 received only nanofat and PRP infiltration | 1. Fat was 1st centrifuged at 3000 rpm for 3 min 2. Emulsification (30 passes) 3. Addition of 3 cc of the patient’s own PRP | Chronic acne scars | Donor site: lower abdomen, flanks, hips and thighs  A 3-mm Coleman aspiration cannula with manually generated negative pressure | Postoperative skin thickness measured with ultrasound scan.  FACE-Q module to analyze satisfaction and aesthetic perception. | NA | 1-month, 3-month and 6-month | - The difference in skin thickness between the 2 groups was not statistically significant - Preoperative and postoperative skin thickness values were compared for each group and showed a significant increase in skin thickness - Postoperative FACE-Q module in both group A and group B found no significant difference |
| 2) Gentile *et al.* 2017 | 43 | Three variants of nanofat:  1: supercharged nanofat  2: evo-nanofat 3: centrifuge-modified nanofat were experimented against Control group: classic nanofat | Control group:  Classic nanofat:   - Fat mechanically emulsified (30 passes) before filtration over a nylon cloth - There are 3 other modified nanofats prepared in different ways: pages 2-4 | burn or post-traumatic scars | Donor site: NA  lipoaspirate obtained with a Coleman cannula (3 mm-diameter) | - Flow cytometry of all nanofat variants - Evaluation of per- and postoperative photographs by authors, - Clinical evaluation by the operators - Self-evaluation by the patients - Histological analysis of biopsy | No significant complications or other unwanted side effects | 3, 4, 6 and 12 months | - Immunophenotypic characterization showed no significant difference between groups - Supercharge-modified nanofat gave the most cell yield followed by evo-, centrifuge-modified and lastly the classic nanofat - Clinical outcomes: team (operators) evaluation and patient self-evaluation indicated that supercharge-modified nanofat gave the best results followed by evo-, centrifuge- and lastly the classic nanofat - Histopathologically; total epidermal and dermal thickness, measured for each experimental group revealed a significant increase in skin thickness at 6 months post treatment - No substantial difference in skin thickness and regeneration between the different nanofat procedures was observed |
| 3) Uyulmaz *et al.* 2018 | 40 | Retrospective analysis study: the study sample received one treatment, and the results were compared with preoperative status | 1. fat was first emulsified (30 passes through 2.4 mm transfer) 2. filtration through a nylon cloth | variable | - Donor site: abdomen and flanks - lipoaspirate was harvested using the Tonnard Harvester 2.4 mm × 20 cm cannula with side holes of 1 mm in diameter | - Patient satisfaction assessment by an interview - Pre- and postoperative photographs were evaluated by three physicians on visual three-grade scale | No significant complications, no fat cysts, infections, foreign body reactions, permanent discolorations, or other side effects | 3 months | - Reviewers classified the results in the majority of scars posttreatment as good 74%, 18% as satisfactory and only 8% as unchanged - 92% of patients were highly satisfied with their results |
| 4) Gu *et al.* 2018 | 20 patients (25 scar site) | Prospective case series: one study group who received nanofat and the results were compared with preoperative status. | 1. Lipoaspirate was 1st centrifuged at 3000 rpm for 3 minutes; 2. then emulsified (30 passes, 1.4 transfer); 3. finally centrifuged again at 3000 rpm for 3 minutes | Atrophic scar: the most common cause was surgical sutures; burns, trauma, and acne | 1. Donor site: paraumbilical region 2. According to the Coleman protocol, liposuction with a 3.0-mm multihole cannula, negative pressure created manually | 1. Patient and Observer Scar Assessment Scale (POSAS) 2. Photographs were taken preoperatively and 6 months postoperatively for comparison 3. Punch biopsies (2 × 2 mm) before and 6 months after and used for immunohistochemical analysis | NA | Not specified | - A significantly improved overall POSAS score among both; patients and observers - Enhancement of staining of melanin in the basal cell layer and a statistically significant increase in melanin between preoperative and postoperative - No difference in staining of elastic fibers between preoperative and postoperative evaluation and no significant change in the fractional area occupied by elastic fibers - No sebaceous glands, or sweat glands were observed preoperatively, but sebaceous and sweat glands appeared postoperatively |
| 5) Jan *et al.* 2019 | 48 | Prospective case series: one study group who received nanofat and the results were compared with preoperative status | Emulsification only without filtration (30 passes) | Postburn scars | - Donor site: abdomen, lateral thigh, or gluteal region - Fat was harvested using a 3-mm cannula with sharp side holes of 1 mm | Patient Observer Scar Assessment Scale (POSAS) | - mild edema (62.5%) - No bruising - No fat cysts or granulomas | 6 months | - Statistically significant improvement in scar quality after use of nanofat - Significant improvements in pliability and pigmentation on the observer scale - A statistically significant improvement in all parameters of the patient section of the POSAS |
| 6) Bhooshan *et al.* 2018 | 34 | Prospective case series: one study group who received nanofat and the results were compared with preoperative status | 1. Emulsification (30-35 passes) 2. Filtration using two‑layered gauze | Scars of varied aetiologies (79.4% post‑traumatic) | - Donor site: lower abdomen - Harvested with a 3 mm mirrored triport Colemans cannula by syringe liposuction technique | Patient Observer Scar Assessment Scale (POSAS) | - Two cases of itching, redness, and excoriation of the scar - Minimal complications | 3 months | - Total patient score showed a significant improvement compared to pre‑operative score - Total observer score showed a significant improvement compared to pre‑operative |
| 7) Huang *et al.* 2021 | 44 | Retrospective study: the study subjects received the same treatment, and the results were compared with preoperative status | 1. Centrifugation at 2000 rpm/min for 3 minutes 2. Emulsification (30-35 passes) 3. Filtration using two‑layered gauze | Depressed facial scars | - Donor site: abdomen or inner thigh - Harvested with a 3 mm cannula with multiple sharp sides under negative pressure | - Patient Satisfaction survey based on the FACE-Q scale - Three plastic surgeons assessed preoperative and postoperative photographs (score 1-4) | - 93%: temporary erythema - 2.3%: pigmentation   until half a year   - 4.5%: blistering around the injection   no other surgical site complications, | 12 months | - FACE-Q: patients who had finished the treatment more than one year showed significantly higher satisfaction with their decision to undergo this therapy - In contrast, patients who had finished the therapy less than one year had significantly higher satisfaction with social function than those more than one year - No significant difference was found in satisfaction with the results or with appearance - Surgeons’ evaluation: complete healing in 30% of cases, obvious improvement in 41%, effective in 20% and only 9% of patients were rated as no change after therapy |
| 8) Rageh *et al.* 2021 | 30 | Prospective case series: one study group who received nanofat and the results were compared with preoperative status | - centrifugation at 1006 g‑force/ 3 min - Emulsification (30 passes, 2.4, 1.4‑ and 1.2‑mm) - Filtration by NanoTransfer 400 and 600‑μm | Scars of varied aetiologies (63.3%post‑traumatic) | - Harvesting through Coleman's technique using a Sorensen harvester connected to a syringe - Negative pressure manually | - patients’ satisfaction using quartile scale - Evaluation by dermatologists using Vancouver scar scale (VSS) - Histopathological evaluation of pre- and posttreatment biopsies | - Bruising (13.3%) - Edema (16.7%) - Erythema (10%) - Hyperpigmentation (3.3%) - Rest of patients showed no complications | 6 months | - VSS: Showed an overall significant improvement after treatment - This was attributed to a significant improvement in height, and pliability of scar - Vascularity and pigmentation had no significant difference in VSS - Histopathological: significant improvement in epidermal thickness, collagen and elastic fibers thickness and neovascularization. no significant difference regarding melanocyte staining |
| 9) Kemaloğlu *et al.* 2021 | 45 | Prospective study   - control group 1: no injection - group 2: fat injection - Group 3: nanofat+fat injection | 1. Centrifugation of fat at 3000 rpm 2. Emulsification (30 passes) 3. Filtration over a sterile nylon cloth | Breast reduction scars | - Donor site: lateral border of the breasts - By using a Coleman harvesting cannula attached to a 10-ml syringe - Negative pressure manually | - Evaluation by independent blinded reviewers using Vancouver scar scale (VSS) - Evaluation by patients using Visual analogue scale | No complications were observed | 6 months | - Except for scar height, all VSS scores in the fat and fat-nanofat groups were significantly lowered (improved) compared to those of the control - When comparing the fat and fat-nanofat groups, pigmentation scores were significantly lower in the fat-nanofat - No significant difference in vascularization, pliability and height score between the fat and fat-nanofat - The VAS scores were significantly lower in the fat and fat-nanofat groups - no statistical difference in VAS scores between the fat and fat-nanofat groups |
| 10) van Dongen *et al.* 2022 | 34 | Prospective, double-blind, placebo-controlled, randomized trial | 1. centrifugation of lipoaspirate at 960 g for 2.5 minutes 2. Emulsification using a 1.4 mm connector (30 times) 3. Centrifugation again at 960 g for 2.5 minutes | Breast reduction scars | - Donor site: abdominal region - Using Tonnard harvesting cannulas | - 1. Patient and Observer Scar Assessment Scale (POSAS)   2. Photographic evaluation using VAS by plastic surgeons   3. Histological analysis of pre- and postoperative biopsies | No complications were observed | 6 months and 1 year | - At 6 months posttreatment, tSVF had significantly improved scar appearance as assessed by the POSAS - No difference was observed at 12 months posttreatment - No improvement was seen based on the evaluation of photographs and histologic analysis of postoperative scars between both groups |
| 11) Ramaut *et al.*2024 | 12 | Split Scar–Controlled, Randomized, double-blind Clinical Trial:  One side of scar treated with nanofat and the control side remained untreated. | - Centrifugation for 1 min at 1200 rpm - Emulsification 30 times through 2.4 mm–diameter connector, then 30 times through 1.2 mm - filtration over a 600 to 400 µm metal grid filter | Sutured surgical wound after abdominoplasty | Donor site: upper abdomen and flanks  A manual negative pressure liposuction  using a 3 mm blunt cannula with sharp side holes | - Patient and Observer Scar Assessment Scale (POSAS) - Histological analyses of post-operative biopsies - Spectrophotometry | No difference in the occurrence of complications between both sides | 3, 6, 8, and 12 months | - Nanofat promotes early clinical scar maturation according to patients and observers but does not significantly improve the final scar appearance - Spectrophotometry revealed improved early scar maturation after nanofat infiltration. No significant difference was found after 1 year - At the 8-month follow-up, tissue biopsy analysis revealed no significant differences in epidermal or dermal thickness, elastic fiber content, collagen organization, or collagen disposition between treated and control scar samples |
| 12) Rageh *et al.* 2025 | 30 | Prospective case series: one study group who received nanofat and the results were compared with preoperative status | - Centrifugation at 3,000 rpm for 3 minutes - Emulsification between 2.4-, 1.4-, and 1.2-mm connectors - Filtration (filter of 400 µm and 600 µm) | Post-burn scars | Abdomen | - Vancouver Scar Scale (VSS) - Antera 3D Camera Analysis - Patient satisfaction survey | No adverse effects | 4 months | - Significant improvement in VSS scores (scar height, pliability, vascularity, pigmentation) after nanofat injections - Antera 3D analysis showed significant reductions in indentation, erythema, and pigmentation - 40% of patients reported good improvement, 33.3% moderate improvement, 13.3% excellent improvement - Nanofat injection proved to be a safe and effective method for post-burn scar treatment |
